# Mechanistic Insights of Aberrant Splicing with Splicing Factor Mutations Found in Myelodysplastic Syndromes

**DOI:** 10.3390/ijms22157789

**Published:** 2021-07-21

**Authors:** Naoyuki Kataoka, Eri Matsumoto, So Masaki

**Affiliations:** 1 Laboratory of Cellular Biochemistry, Department of Animal Resource Sciences, Graduate School of Agriculture and Life Sciences, The University of Tokyo, Tokyo 113-8657, Japan; matsumoto.eri@mail.u-tokyo.ac.jp; 2 Laboratory of Molecular Medicinal Science, Department of Pharmaceutical Sciences, Ritsumeikan University, Shiga 525-8577, Japan; smasaki@fc.ritsumei.ac.jp

**Keywords:** MDS, splicing, splicing factor mutations, exon recognition

## Abstract

Pre-mRNA splicing is an essential process for gene expression in higher eukaryotes, which requires a high order of accuracy. Mutations in splicing factors or regulatory elements in pre-mRNAs often result in many human diseases. Myelodysplastic syndrome (MDS) is a heterogeneous group of chronic myeloid neoplasms characterized by many symptoms and a high risk of progression to acute myeloid leukemia. Recent findings indicate that mutations in splicing factors represent a novel class of driver mutations in human cancers and affect about 50% of Myelodysplastic syndrome (MDS) patients. Somatic mutations in MDS patients are frequently found in genes *SF3B1, SRSF2*, *U2AF1*, and *ZRSR2*. Interestingly, they are involved in the recognition of 3′ splice sites and exons. It has been reported that mutations in these splicing regulators result in aberrant splicing of many genes. In this review article, we first describe molecular mechanism of pre-mRNA splicing as an introduction and mainly focus on those four splicing factors to describe their mutations and their associated aberrant splicing patterns.

## 1. Introduction

### 1.1. Splicing Signals and Splicing Reaction

**Figure 1:**
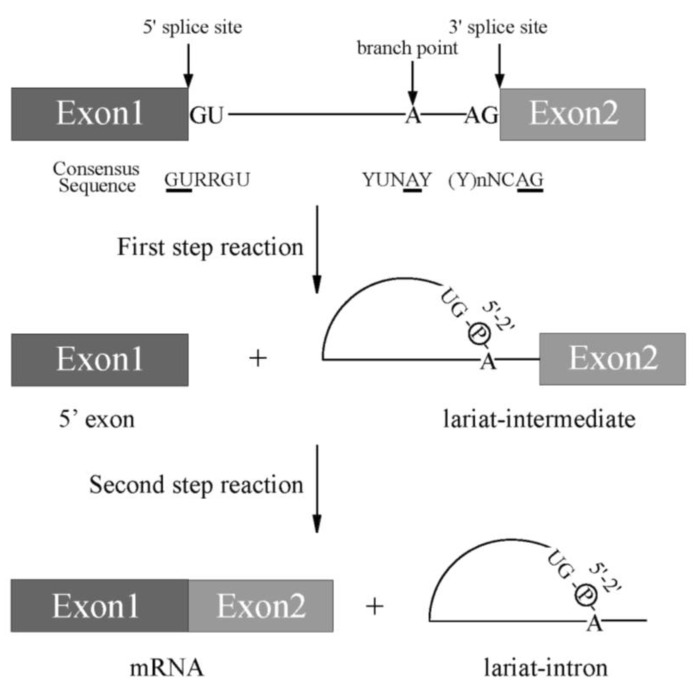
A scheme for splicing reaction with two steps. Schematic representation of sequences required for splicing reaction. Boxes show exons, and lines between boxes represent introns. Conserved sequence elements of metazoan pre-mRNAs. R and Y stand for purine and pyrimidine residues, respectively. N indicates any nucleotides. Conserved 5′ and 3′ splice sites, and Adenosine residue used for branch nucleotide are underlined.

Pre-mRNA splicing is a critical step for protein expression in higher eukaryotes [1]. For constitutive splicing, all exons are ligated in order without any insertions and deletions of nucleotides. The essential signals for splicing reaction mostly reside at both ends of introns (Figure 1) [1]. At the 5′ end, consensus sequence of GURRGU (R stands for purine) can be found in most of introns in mammals (Figure 1). This site is called 5′ splice site (5′ss). CAG consensus sequence is often discovered at the 3′ end of introns (Figure 1), which is called 3′ splice site (3′ss). In addition to them, pyrimidine (Y) residue stretch precedes to 3′ splice site in order to support recognition of 3′ splice site in mammals (Figure 1, (Y)nNCAG). A branch point sequence (BP), at which lariat formation occurs by 2′–5′ phosphodiester bond formation with Guanine residue at 5′ splice site, resides 20–30 nucleotides upstream of the 3′ splice site (Figure 1). Although the sequence for branch point in budding yeast is well-conserved as UACUAAC (underlined A is a branch point) among introns, the conserved sequence around branch point in mammals is YUNAY (branch point is underlined, Y and N stand for pyrimidine and any nucleotide, respectively), which is more diverse (Figure 1) [2]. Then, pyrimidine residue stretch also supports branch point sequence recognition (Figure 1). The splicing reaction consists of two steps, the first step and the second step. In the first step reaction, cleavage at 5′ ss and formation of lariat structure in intron occur. The second step reaction includes cleavage at 3′ ss and ligation of exons to produce mRNA. Both steps require ATP and divalent cations in vitro. As a divalent cation, magnesium is most efficient in in vitro splicing reaction.

### 1.2. Spliceosome Formation with Splicing Factors

**Figure 2:**
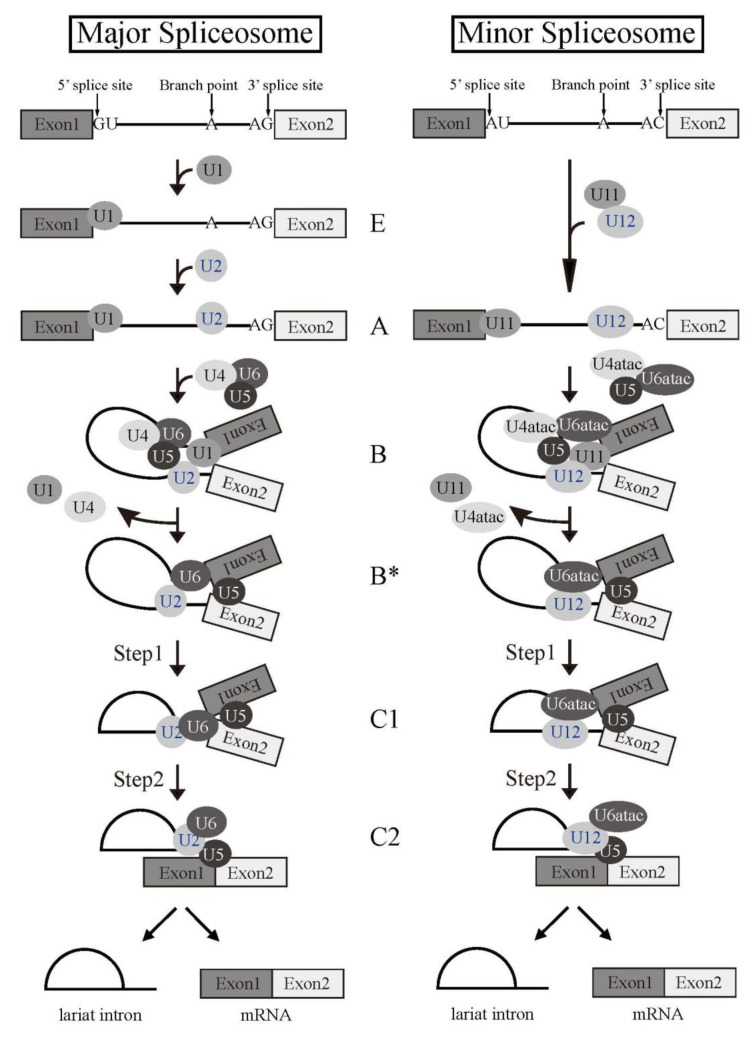
Splicing reaction and formation of spliceosome. Schematic representations of major and minor spliceosome formations. Both splicing reactions take place stepwise in a spliceosome. Spliceosomal Uridine-rich small nuclear ribonucleoproteins (U snRNPs) are indicated with their names. The name of each spliceosome intermediate complex is shown in the middle.

Splicing reaction takes place in a large ribonucleoprotein complex, termed the spliceosome [1]. The assembly of the spliceosome on pre-mRNA occurs with stepwise association of the uridine (U)-rich small nuclear RNPs (snRNPs) (U1, U2, U4, U5, and U6) (Figure 2) and a multitude of non-snRNP splicing factors [1]. U snRNPs consist of short RNA, Sm proteins, and a few specific proteins of each U snRNPs. As the first step of the reaction, 5′ splice site is recognized by U1 snRNP by RNA-RNA pairing. U2 snRNP then come to associate with a branch point sequence with the help of U2 snRNP auxiliary factor (U2AF) complex that consists of U2AF1 and U2AF2 heterodimer. The RNA component of U2 snRNP also hybridizes with pre-mRNA to recognize BP. The tri-snRNP, U4/U5/U6, then becomes joining to the spliceosome. Two U snRNPs, U4 and U6, form a heterodimer by pairing their RNA components. The spliceosome is activated by removal of U1 and U4 snRNPs to remodel pre-mRNA-U snRNPs and U snRNP-U snRNP interactions, and the first step reaction, the cleavage at the 5′ splice site and formation of a lariat structure, takes place. Then, the cleavage at the 3′ splice site and ligation of two exons occur as the second step reaction. Several lines of evidence suggested that U6 snRNA has catalytic activity for the splicing reaction.

There is another type of intron which is called a minor intron or ATAC intron, since AT and AC are often found at 5′ and 3′ splice sites in genome DNA, respectively (Figure 2). There are about 800 minor introns, which corresponds to 0.4% of total introns in humans [3,4]. Although it is thought that splicing efficiency of minor introns is less than that of major introns, minor introns remain residing in the genome of higher eukaryotes. It is possible that regulation of gene expression through regulation of minor intron splicing is important for those organisms. For this minor intron spliceosome formation, there is a different set of U snRNPs (U11, U12, U5, U4atac, and U6atac, Figure 2) [3,4]. The 5′ splice site and the branch point sequence is recognized by a heterodimer that consists of U11 snRNP and U12 snRNP, respectively. Instead of U4 and U6 snRNPs, U4atac and U6atac snRNPs are recruited to a minor spliceosome. Interestingly, U5 snRNP is a common snRNP for both major and minor introns (Figure 2). For both major and minor spliceosomes, formation of the spliceosome takes place with stepwise assembly of complexes designated as H, E, A, B, and C (Figure 2). The spliceosome is dissociated into two complexes, the mRNP complex and the intron complex, by an RNA helicase RHX34/HRH1 [5]. The mRNP complex, which contains Exon Junction Complexes on mRNA [6,7], will be exported to the cytoplasm, and mRNAs will serve as templates for translation. On the other hand, the intron complex is supposed to be retained and degraded in the nucleus after the removal of U snRNPs and other splicing factors. Post-splicing intron complexes are degraded via the Intron Large (IL) and Intron Small (IS) complexes [8]. The IL complex contains U2, U5, and U6 snRNPs, whereas those U snRNPs are barely detected in the IS complex [8]. The IL complex also contains hPrp19 complex factors whose homologs in budding yeast are involved in both mRNA splicing and the DNA repair process [8]. Those findings suggest an interplay between mRNA splicing and transcription-coupled DNA repair (TCR). Removal of U snRNPs from the IL complex is mediated by the hPrp43/TFIP11 complex in humans [8]. After dissociation of splicing factors, introns in the IS complex are susceptible to RNA lariat debranching enzyme 1 (Dbr1) protein that linearizes introns via dissolving 2′–5′ phosphodiester bond [8,9]. Interestingly, hDbr1 can shuttle between the nucleus and the cytoplasm [10], suggesting that it is involved in the RNA quality control process by linearizing intron-lariat containing RNAs accidentally exported from the nucleus.

### 1.3. Alternative Splicing

**Figure 3:**
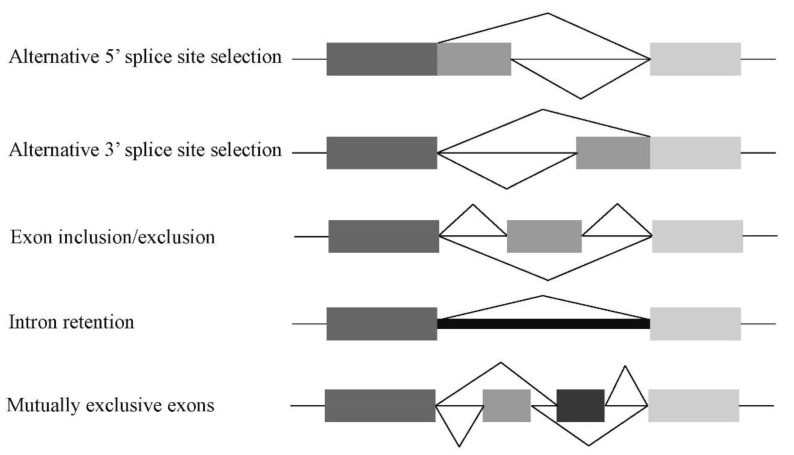
Major patterns of alternative splicing. Schemes of five major alternative splicing patterns in higher eukaryotes. In intron retention alternative splicing, the intron shown in a thick line is recognized as an alternative exon.

The splicing described above is termed constitutive splicing, which utilizes all exons in the pre-mRNA molecule. In contrast, another type of splicing, alternative splicing, employs several alternative exons for both inclusion and exclusion [11,12]. There are several types of alternative splicing, such as alternative 5′ or 3′ splice sites, exon-skipping/-inclusion, intron retention, and mutually exclusive exons (Figure 3). Alternative splicing produces many variants of mRNAs that are translated into proteins with different structures and functions. Therefore, this step likely contributes to generating diversity of the expressed proteins in higher eukaryotes from the limited number of genes [13]. The Human Genome Project also revealed that over 95% of human genes undergo alternative splicing [14,15]. Abnormalities of both constitutive and alternative splicing in humans may cause dysfunctions or absence of the encoded functional proteins, often resulting in hereditary diseases [16].

### 1.4. Cis-Regulatory Elements and Trans-Acting Factors in Splicing

**Figure 4:**
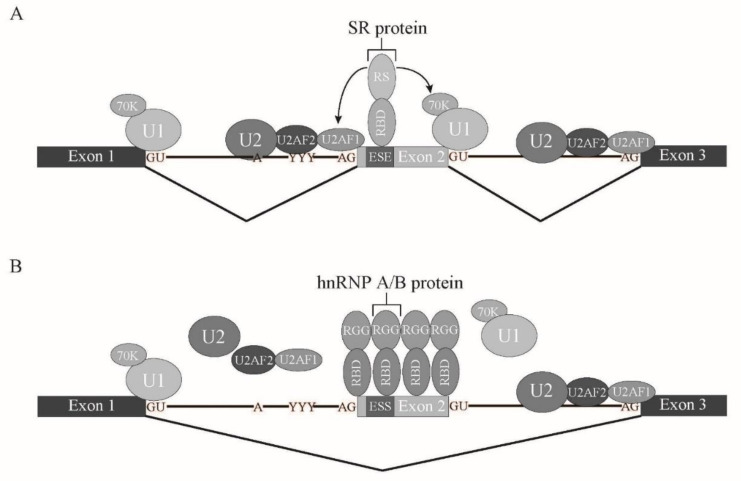
Models for promotion or inhibition of exon recognition by SR proteins or hnRNP A/B proteins, respectively. (**A**) SR proteins bind to exonic splicing enhancer (ESE) through their RNA binding domain (RBD). They interact with U2AF1 and U1 70K proteins via interactions between their Arginine-Serine-rich (RS) domains, which promotes exon recognition. U1, U2: U1, U1 small nuclear ribonucleoprotein, U2 small nuclear ribonucleoprotein. U2AF1, U2AF2: U2 snRNP auxiliary factor 1, 2. (**B**) hnRNP A/B proteins bind to the exonic splicing silencer (ESS) via their RBD and can spread on pre-mRNA in both 5′ and 3′ directions. This results in covering the exon and other regions on pre-mRNA and prevents association of splicing factors. Consequently, this exon tends to be excluded. RGG: Arginine-Glycine-Glycine repeat-rich domain.

In vertebrates, intron length is much longer than that in lower eukaryotes. Thus, it is assumed that exon recognition, rather than intron recognition, is a major mechanism for splicing [11,17]. For exon recognition, both upstream and downstream intronic regions of the corresponding exon play important roles. The upstream intronic region includes the branch point (BP), polypyrimidine stretch, and 3′ splice site AG dinucleotide, while the downstream intron region has a 5′ splice site sequence. BP is recognized by U2 snRNP in accordance with the SF3B complex [1,18,19]. Polypyrimidine stretch and AG dinucleotide are bound to U2AF2 and U2AF1, respectively, which form a heterodimer [1,18,19]. The 5′ splice site downstream of exon is associated with U1 snRNP. In addition to those intronic elements, some exons contain exonic regulatory elements that are called exonic splicing enhancer (ESE). ESE is often bound to Serine-Arginine-rich splicing factor (SRSF) family proteins to promote exon recognition [18,20,21]. To date, twelve SR proteins have been found in humans [21]. SRSF family proteins share a common feature, one or two RNA binding domains (RBDs) at amino-terminus and Arginine-Serine-rich (RS) domain at carboxy terminus [18,20,21]. RS domain is a protein-protein interaction domain among RS domain-containing proteins [18,20,21]. U2AF1/U2AF35, U2AF2/U2AF65, and U170K, a component of U1 snRNP, also harbor the RS domain. SRSF proteins bind to ESE by its RBD and interact with both U2AF and U170K through the RS domain, which bridges the 3′ splice site and 5′ splice site over the exon (Figure 4). This crosstalk between 3′ and 5′ splice sites promotes the recognition of the exons that have ESEs [11,17]. In contrast, there is another regulatory sequence that reduces exon recognition. This regulatory element is called exonic splicing silencer (ESS). ESSs are bound to another class of splicing regulators, heterogeneous nuclear ribonucleoproteins (hnRNPs). The hnRNP protein family consists of 20 proteins, named from A1 to U, which have many different RNA binding domains [22]. hnRNPs are nuclear abundant proteins that have many cellular functions, such as transcription, splicing, RNA transport/localization, translation, and RNA stability [22]. It was demonstrated that one of the hnRNP family proteins, hnRNP A1, mediates silencing by binding initially to a high-affinity binding site in the exon, which then promotes further hnRNP A1 association with the upstream and downstream regions of the exon [23]. Thus, this results in inhibition of ESE binding of SR proteins (Figure 4). The elements described above are also found in introns, which, in turn, are called Intronic Splicing Enhancer (ISE) and Intronic Splicing Silencer (ISS) [11]. Those regulatory elements and regulatory factors are involved not only in constitutive splicing, but also in tissue- and stage-specific alternative splicing [11]. Both SR proteins and hnRNP proteins are involved in alternative splicing. SR proteins tend to promote exon inclusion by binding to ESE and ISE, whereas hnRNP proteins, especially hnRNP A/B proteins, have a tendency to enhance exon skipping via binding to ESS and ISS [11]. The ratio between SR protein and hnRNP A/B protein levels varies among tissues [24], which likely contributes to tissue-specific alternative splicing. Several tissue-specific splicing regulators are also reported. For example, Nova, KSRP, and RbFox1 proteins are neuron-specific RNA binding proteins that mediate neuron-specific alternative splicing [11]. RNA binding motif protein 24 (Rbm24) and Rbm38 are required for muscle differentiation through splicing modulation [11]. Epithelial cells also express epithelial-specific splicing regulators, ESRP1 and 2, to pursue epithelial-specific alternative splicing [11]. It is likely that the combination of expression levels of SR proteins, hnRNP proteins, and tissue-specific splicing regulators determines which alternative splicing pattern is selected in each tissue.

### 1.5. Human Diseases Caused by RNA Processing Defects

Mutations in those *cis*-regulatory elements and *trans*-acting splicing regulators cause aberrant splicing patterns that often result in diseases in humans. These diseases that have defects in RNA metabolism steps have recently been called ‘RNA diseases’, and it is estimated that up to 15% of all point mutations that result in human genetic disease cause an RNA splicing defect [16]. Ten years ago, it was demonstrated that splicing factors are frequently mutated in myelodysplastic syndrome (MDS). MDS is a heterogeneous group of chronic myeloid neoplasms characterized by many symptoms, such as ineffective hematopoiesis, peripheral blood cytopenia, and a high risk of progression to acute myeloid leukemia [25]. Next-generation sequencing of the patients’ genome DNA revealed that SF3B1, U2AF1, SRSF2, and ZRSR2 are the most frequently mutated splicing factors [26,27,28]. Since SF3B1, U2AF1, and SRSF2 have the particular amino-acid residues mutated in many patients, those mutations are supposed to cause ‘gain-of function’ mutations in those genes. In contrast, many different mutations were found in ZRSR2 genes, suggesting that those mutations result in ‘loss-of-function’ of the ZRSR2 gene product. Therefore, it was assumed that aberrant splicing caused by splicing factor mutations results in onset of MDS. Several lines of evidence have been accumulated to demonstrate how MDS onsets are caused by mutations in certain splicing factors.

In this review article, we mainly introduce four of the most frequently mutated splicing factor genes (SF3B1, U2AF1, SRSF2, and ZRSR2) in MDS with the aberrant splicing mechanism caused by those mutations and an outline of MDS from a splicing point of view.

## 2. Splicing Factors Mutated in Myelodysplastic Syndrome

### 2.1. SF3B1

**Figure 5:**
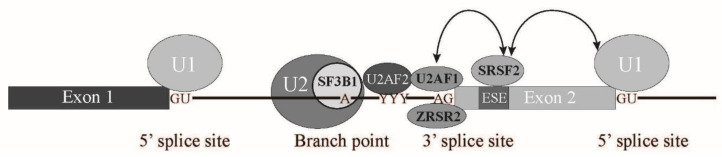
A scheme for early splicing step with splicing factors mutated in MDS on pre-mRNA. The names of four splicing factors whose mutations were frequently observed in Myelodysplastic syndrome (MDS) are shown in black bold. letters. Interestingly, these splicing factors are involved in 3′ splice site and exon recognition during splicing reaction. Arrows indicate protein-protein interaction through an Arginine-Serine-rich (RS) domain. ESE: exonic splicing enhancer, U1, U2: U1 small nuclear ribonucleoprotein, U2 small nuclear ribonucleoprotein.

SF3B1 is one of the components of the SF3B complex that stabilizes U2 snRNP binding to the branch point sequence during pre-mRNA splicing (Figure 5) [2,29]. SF3B1 gene is located on chromosome 2q33.1. Approximately 20–28% of MDS patients harbor SF3B1 mutations [27,30,31,32]. Surprisingly, SF3B1 mutations are responsible for the ring sideroblast (RS) phenotype in ∼98% of cases [31]. In a study with NOD scid gamma (NSG) mouse, mice transplanted with hematopoietic stem cells (HSCs) from SF3B1 mutant MDS-RS patients develop the characteristic ring sideroblasts phenotype [33]. Most recently, the International Working Group for the Prognosis of Myelodysplastic Syndromes (IWG-PM) provided supporting evidence that shows the recognition of SF3B1-mutant MDS as a distinct diagnostic entity [34]. The dataset they used includes 3479 patients with known SF3B1 mutation status that represents the largest MDS data set with genetic data reported to date. Their validation strongly supports the correlation of SF3B1 mutations with clinical phenotype in MDS. SF3B1 mutations in MDS patients have a cluster as a ‘hot spot’ at 700th residue of Lysine changed to Glutamine, which resides in HEAT domain repeats [32,35]. Other hotspots (R625, H662, and K666) are also assumed to have a similar functional impact due to their close spatial proximity in HEAT repeats [32,36]. Many studies have demonstrated that SF3B1 mutations cause aberrant splicing via cryptic 3′ splice site usage [37,38,39,40,41]. Since the SF3B complex is involved in recognition of a branch point sequence, this mutation is highly likely to cause cryptic branch point sequence recognition and usage of 3′ splice site. The aberrant splicing caused by cryptic 3′ splice site usage often creates premature termination codons in the mRNA, resulting in transcript degradation by nonsense-mediated decay (NMD) [42,43]. In SF3B1 mutant samples, reduction of intron-retaining isoforms was consistently reported [44,45]. These results suggest that reduced intron retention is due to the ability of SF3B1 mutants to select an upstream aberrant 3′ splice site [44]. Decreased intron retention was more prominent in the cytoplasm of SF3B1 mutant cells, suggesting that nuclear export of intron-retaining transcripts was impaired [45]. By using RNA-sequencing, many dysregulated gene isoforms and aberrantly spliced target genes in SF3B1 mutant MDS have been identified [37,39,40,46,47,48,49,50]. It becomes of great interest to identify and functionally characterize specific target genes of mutant SF3B1 in order to discover drug targets. As for drug candidates, it has been suggested that SF3B1 inhibitors have potential in treating the preleukemic state and related myeloid disorders. Since SF3B1 is essential for splicing, it was expected that SF3B1 inhibitors completely block the splicing reaction for all introns. However, inhibition of splicing by spliceostatin A (SSA) is partial in cultured cells and produces shorter transcripts that are translated into truncated proteins in tumor cells [51,52]. Another compound, pladienolide B, is an antitumor macrolide, and it was also found to interact with SF3b to inhibit splicing [53]. E7107, a derivative of pladienolide D, displayed strong antitumor activity [53]. It turned out that E7107 blocks spliceosome assembly by preventing tight binding of U2 snRNP to pre-mRNA [52]. It is worthy to design small chemical compounds that strongly associate and inhibit SF3B1 as drug candidates for MDS and other cancers such as breast cancer and lung adenocarcinoma.

### 2.2. SRSF2

SRSF2, originally called SC35 [54], is a member of the SR protein family that is involved in both constitutive and alternative splicing [11,21]. The SRSF2 gene is located on chromosome 17q25.2. SRSF2 mutations have been found in about 14% of patients with MDS [55]. SRSF2 contains an RBD for RNA binding and an RS domain for interaction with other proteins. SRSF2 promotes exon recognition by binding to ESE in pre-mRNA through its RBD (Figure 5). Through its RS domain, SRSF2 interacts with U2AF heterodimer and U170K, which results in promotion of the association of those factors to the upstream 3′ splice site and the downstream 5′ splice site, respectively [11,18,56]. In MDS patients, the mutations in SRSF2 are clustered in the 95th Proline residue as a ‘hot spot’ [28,57]. Like SF3B1 and U2AF mutations in MDS, it was assumed that these hot spot mutations cause gain-of function of the mutant proteins. Since this Proline residue resides slightly outside of the RNA Binding Domain of SRSF2, it was assumed that the mutations in MDS do not affect RNA binding activity of SRSF2. However, splicing pattern changes with SRSF2 mutations were reported in culture cells, mouse models, and primary human samples [57,58,59]. Analyses and comparison of binding sequence motifs for wild type and mutant SRSF2 proteins revealed that mutant SRSF2 proteins have higher binding affinity to GGNG and CCNG motifs (C as Cytosine, G as Guanosine, N as any nucleotides) in addition to the Purine-rich motif, which wild type binds efficiently [57,58,59,60]. This affinity change results in differential splicing of many genes, including EZH2, a gene implicated in the pathogenesis of MDS [61]. EZH2 is a SET-domain containing histone methyltransferase that is a component of the Polycome Repressive Complex 2 (PRC2). PRC2 catalyzes tri-methylation of histone H3 at Lys 27 (H3K27me3) to regulate gene expression. Since aberrant splicing of EZH2 under mutant SRSF2 includes exon 9.5 that contains the stop codon, protein level of EZH2 is likely reduced in MDS patient cells [45,57,62,63]. Another epigenetic factor mutation implicated a pathogenic crosstalk between altered states of epigenome and splicing in a subset of leukemias. Yoshimi et al. demonstrated that aberrant splicing of INTS3 contributed to leukemogenesis in concert with mutant IDH2 and was dependent on mutant SRSF2 binding to cis-regulatory elements in INTS3 pre-mRNA and increased DNA methylation of INTS3 [64].

It has also been demonstrated that MDS-responsible mutations in SRSF2 and U2AF1 cause expansion of R loop [65,66]. Expansion of R loop formation results in activation of the DNA damage response pathway [66]. Efficient formation of R loop may take place by slowing down rearrangement of mRNA-protein complexes during/after splicing. It is of great interest how splicing factors take part in R loop formation and/or resolution.

### 2.3. U2AF1

U2AF1 is originally identified as a component of the U2 snRNP auxiliary factor complex (U2AF) that facilitates association of U2 snRNP to the branch point sequences [67,68]. U2AF1 is a small subunit of the U2AF heterodimer that is responsible for the recognition of AG dinucleotide in pre-mRNA 3′ splice sites (Figure 5) [1,11,18]. Another subunit, U2AF2, recognizes a pyrimidine stretch residing between the branch point sequence and the 3′ splice site [67,68]. Since U2AF1 and 2 contain the RS domain, they can interact with the U170K protein that also has an RS domain to get an interaction between 3′ splice site and downstream 5′ splice site over the exon. The U2AF1 gene is located on 21q22.3, and its mutations occur in approximately 7–11% of MDS patients [28,30,32,69]. U2AF1 mutations also have ‘hot spots’ at S34 and Q157 that are located in conserved zinc finger domains, Zn1 and Zn2 [26,30,32,70,71]. Both S34 and Q157 mutations in U2AF1 have been shown to affect splicing through RNA binding activity, but they have different effects on 3′ splice site recognition. It was demonstrated that U2AF1 S34 mutants tend to promote aberrant exon inclusion when the 3′ splice site sequence is CAG or AAG [71,72,73,74,75,76,77]. In contrast, Q157 mutants affect recognition at one nucleotide downstream of the 3′ splice site AG dinucleotide, promoting exon inclusion when a Guanine is at this position [73]. Recently, it was found that U2AF1 S34 mutant induces inclusion of exon 4 in alternative splicing of interleukin-1 receptor-associated kinase 4 (IRAK4). This isoform encodes IRAK4-L protein that causes innate immune activation [78]. IRAK4 activates NF-κB and MAPK pathways via mediating signaling downstream of the Toll-like receptor (TLR) superfamily [78]. IRAK4 is divided into two spliced isoforms dependent on exon 4 being contained or excluded: IRAK4-L and IRAK4-S [78]. IRAK4-S could control the innate immune response in normal hematopoietic cells, while IRAK4-L mediates NF-κB maximal activation, resulting an uncontrolled innate immune response in malignant hematopoietic cells [78]. IRAK4-L is also expressed highly in breast and colon cancer cell, indicating its association with oncogenicity [78]. Furthermore, mutant U2AF1(S34F) AML cells acquire a dependency on IRAK4-L and are sensitive to IRAK4 inhibitors, which suggests a therapeutic strategy [78]. Most recently, crystal structure analysis of yeast U2AF1 revealed that the 3′ splice site AG dinucleotide is strongly recognized by the two Zn finger domains and how aberrant alternative splicing occurs with MDS mutations [79]. By using Förster resonance energy transfer (FRET), the influence of both wild-type or S34F mutant U2AF1 on the conformational dynamics of U2AF2 and RNA complexes was also determined. Warnasooriya et al. demonstrated that the U2AF heterodimer (U2AF1 + U2AF2) binds weak pyrimidine tracts as a mixture of closed and open U2AF2 conformations, and the S34F mutation of U2AF1 modulates shifts between open and closed U2AF2 [80]. It may help to design chemical compounds targeting mutated Zn finger domains to inhibit or modify their RNA binding activity as therapeutic approaches to MDS.

### 2.4. ZRSR2

The ZRSR2 gene is located on chromosome Xp22.2 and mutated in about 5% of MDS patients, predominantly males [28]. Relatively little is known about this protein’s function in mRNA splicing. This protein is another member of the SR-rich family of splicing factor, and it was shown to be responsible for the recognition of the 3′ splice acceptor site for both a major and a minor intron in vitro (Figure 5) [81]. Since the mutations of ZRSR2 in MDS patients were found all over the coding region as out-of-frame insertions, deletions, nonsense, and missense, the nature of the mutations is likely loss-of-function [82]. The *ZRSR2* mutations cause abnormal splicing via intron retention of U12-depedent minor introns [82]. Humans have a limited number (about 800, 0.4% of total numbers of introns) of minor introns [3,4]. Among them, several genes including some E2F transcription factors and several genes in the MAPK/ERK pathway show aberrant splicing in ZRSR2 mutant MDS samples [82].

Most recently, it was demonstrated that impaired minor intron excision by knock-out of ZRSR2 protein enhances hematopoietic stem cell self-renewal, and mutations in minor introns are suggested to be potential cancer drivers [83]. However, the precise molecular mechanism how ZRSR2 mutations affect minor intron splicing remains to be elucidated. Elucidation of the molecular mechanism for ZRSR2 involvement in minor intron splicing may provide useful information for both basic knowledge for minor intron splicing and identification of targets for MDS with ZRSR2 mutations.

### 2.5. Other Splicing Factor Mutations in MDS

In addition to four splicing factors, rare mutations in several other splicing factors were also identified. One of the mutated splicing factors is PRPF8. Studies from the yeast Prp8 protein revealed that PRPF8 protein is an essential factor for splicing and interacts with U5 snRNA to align 5′ and 3′ splice sites in the spliceosome [84]. PRPF8 mutation causes missplicing [85], highly likely through alteration of splice sites selection. An analysis for the precise mechanism of how PRPF8 mutations affect exon recognition remains to be performed. Another factor is *LUC7L2.* LUC7L2 is an ortholog of splicing factor LUC7 which is involved in recruitment of splicing factors. The *LUC7L2* protein is assumed to be involved in the recognition of non-consensus splice donor sites in association with the U1 snRNP [86]. Interestingly, one RNA helicase protein, DDX41, was shown to be mutated in MDS and AML [87]. Several RNA helicases are known to be involved in splicing steps likely by causing spliceosome conformation change with their ATP-dependent helicase activity. Indeed, the most common mutation is the R525H mutation, which is assumed to affect adenosine triphosphate (ATP) binding [87]. Very rare mutations have also been found in SF3A1, SF1, PRPF40B, and U2AF2 [88], which are mainly involved in recognition of the branch point and the 3′ splice site. It is of great interest for RNA scientists to investigate how these mutations affect splicing reaction. Those analyses will shed light not only on MDS pathogenesis but also on understanding the basic splicing mechanism.

## 3. Conclusions and Future Perspectives

**Figure 6:**
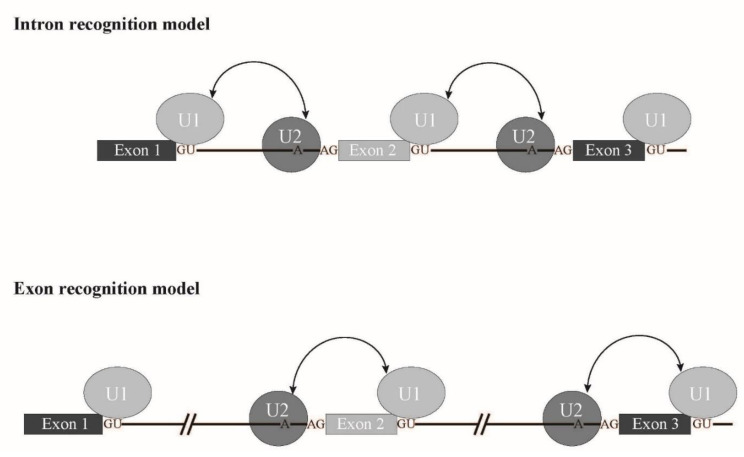
Schematic representation of exon recognition and intron recognition models during splicing. In lower eukaryotes, whose intron size is small, intron recognition is a dominant mode for splicing (upper panel). Introns are recognized by crosstalk between U1 snRNP and U2 snRNP that bind to the 5′ splice site and a branch point, respectively. On the other hand, exon recognition is major for splicing in vertebrates, in which introns are long. In this type, exons are recognized via interaction over exons between U2 snRNP and U1 snRNP that bind to a branch point and the 5′ splice site, respectively (lower panel).

In this review, we introduced four major splicing factors mutated in MDS with aberrant splicing caused by mutations. Interestingly, most of the proteins described in this review are involved in 3′ splice site recognition (Figure 5). In addition, SRSF2 is involved in exon recognition through ESE binding. Taken together, it is likely that a splicing mode called exon recognition (Figure 6) [17] participates in aberrant splicing in MDS. In higher eukaryotes, the average length of introns is much longer than that of lower eukaryotes. In fact, the average length of human introns is 5849 nucleotides, while that of nematodes is 335 nucleotides [89]. In contrast, the average length of internal exons, which is no longer than 300 nucleotides, does not differ between vertebrates and lower eukaryotes. Therefore, exon recognition is likely a major mode for splicing in vertebrates whose intron size is large, while intron recognition is dominant in lower eukaryotes in which introns are relatively short (Figure 6). The facts that 5′ splice site mutations result in skipping of adjacent exons and cause human diseases also support the exon recognition model. For 5′ terminal exons, it is assumed that the cap structure serves as a substitute of the 3′ splice site. The cap structure is recognized by a nuclear cap binding protein complex that consists of NCBP1/2 proteins in the nucleus [90,91,92]. As NCBP1 was demonstrated to associate with U2 snRNP [92], it is possible that NCBP1-U2 snRNP interacts with U1 snRNP at the 5′ splice site to define the first exon. As for 3′ terminal exons, poly(A) addition signal and poly(A) addition machinery are assumed to serve as interactors with U2 snRNP on the branch point in the last intron. As supporting evidence, mutation of the 3′ splice sites inhibits the polyadenylation cleavage reaction in vitro [93]. In the exon recognition model, definition of the 3′ splice site region highly likely takes place first, and this step is critical for exon recognition. Although many excellent works have been performed and provide information for the mechanism of vertebrate exon recognition, it remains unclear whether different factors/mechanism are involved in different exons. It is expected that precise analyses of the aberrant splicing mechanism in MDS with mutant splicing factors also contribute to uncovering the regulation of alternative splicing through exon recognition in vertebrates.

Although some aberrant splicing patterns in dysregulated genes have been identified to be involved in MDS onset as described above, it is still under investigation how different mutations in different splicing factors cause different MDS phenotypes. To date, there seems to be no common gene(s) whose aberrant splicing is responsible for MDS onset caused by mutations in four main splicing factors SF3B1, SRSF2, U2AF1, and ZRSR2. It is assumed that hot spot mutations among them in SF3B1, SRSF2, and U2AF1 do not cause reduction of the encoded proteins, whereas mutations in ZRSR2 reduce functional protein amount. Splicing pattern analyses implicate that common pathways affected by mutations of those factors are epigenetics and signal transduction pathways. These points have to be addressed in future analyses. The approaches from mechanistic analyses of aberrant splicing caused by mutated splicing factors should shed light on research for therapies of MDS by identifying drug targets.

## References

[1] Wahl M.C., Will C.L., Lührmann R. (2009). The Spliceosome: Design Principles of a Dynamic RNP Machine. Cell.

[2] Gao K., Masuda A., Matsuura T., Ohno K. (2008). Human branch point consensus sequence is yUnAy. Nucleic Acids Res..

[3] Patel A.A., Steitz J.A. (2003). Splicing double: Insights from the second spliceosome. Nat. Rev. Mol. Cell Biol..

[4] Verma B., Akinyi M., Norppa A., Frilander M.J. (2018). Minor spliceosome and disease. Semin. Cell Dev. Biol..

[5] Ohno M., Shimura Y. (1996). A human RNA helicase-like protein, HRH1, facilitates nuclear export of spliced mRNA by releasing the RNA from the spliceosome. Genes Dev..

[6] Le Hir H., Sauliere J., Wang Z. (2016). The exon junction complex as a node of post-transcriptional networks. Nat. Rev. Mol. Cell Biol..

[7] Dreyfuss G., Kim V.N., Kataoka N. (2002). Messenger-RNA-binding proteins and the messages they carry. Nat Rev. Mol. Cell Biol..

[8] Yoshimoto R., Kataoka N., Okawa K., Ohno M. (2009). Isolation and characterization of post-splicing lariat–intron complexes. Nucleic Acids Res..

[9] Mohanta A., Chakrabarti K. (2021). Dbr1 functions in mRNA processing, intron turnover and human diseases. Biochimie.

[10] Kataoka N., Dobashi I., Hagiwara M., Ohno M. (2013). hDbr1 is a nucleocytoplasmic shuttling protein with a protein phosphatase-like motif essential for debranching activity. Sci. Rep..

[11] Fu X.-D., Ares M. (2014). Context-dependent control of alternative splicing by RNA-binding proteins. Nat. Rev. Genet..

[12] Lee Y., Rio D.C. (2015). Mechanisms and Regulation of Alternative Pre-mRNA Splicing. Annu. Rev. Biochem..

[13] Nilsen T.W., Graveley B.R. (2010). Expansion of the eukaryotic proteome by alternative splicing. Nature.

[14] Pan Q., Shai O., Lee L.J., Frey B.J., Blencowe B.J. (2008). Deep surveying of alternative splicing complexity in the human transcriptome by high-throughput sequencing. Nat. Genet..

[15] Wang E.T., Sandberg R., Luo S., Khrebtukova I., Zhang L., Mayr C., Schroth G.P., Burge C.B. (2008). Alternative isoform regulation in human tissue transcriptomes. Nature.

[16] Krawczak M., Reiss J., Cooper D.N. (1992). The mutational spectrum of single base-pair substitutions in mRNA splice junctions of human genes: Causes and consequences. Hum. Genet..

[17] Berget S.M. (1995). Exon Recognition in Vertebrate Splicing. J. Biol. Chem..

[18] Kataoka N. (2017). Modulation of aberrant splicing in human RNA diseases by chemical compounds. Hum. Genet..

[19] Scotti M.M., Swanson M.S. (2016). RNA mis-splicing in disease. Nat. Rev. Genet..

[20] Howard J.M., Sanford J.R. (2015). The RNAissance family: SR proteins as multifaceted regulators of gene expression. Wiley Interdiscip. Rev. RNA.

[21] Manley J., Krainer A.R. (2010). A rational nomenclature for serine/arginine-rich protein splicing factors (SR proteins). Genes Dev..

[22] Geuens T., Bouhy D., Timmerman V. (2016). The hnRNP family: Insights into their role in health and disease. Hum. Genet..

[23] Zhu J., Mayeda A., Krainer A.R. (2001). Exon Identity Established through Differential Antagonism between Exonic Splicing Silencer-Bound hnRNP A1 and Enhancer-Bound SR Proteins. Mol. Cell.

[24] Hanamura A., Caceres J.F., Mayeda A., Franza B.R., Krainer R.A. (1998). Regulated tissue-specific expression of antagonistic pre-mRNA splicing factors. RNA.

[25] Cazzola M., Della Porta M.G., Malcovati L. (2013). The genetic basis of myelodysplasia and its clinical relevance. Blood.

[26] Graubert T., Shen D., Ding L., Okeyo-Owuor T., Lunn C.L., Shao J., Krysiak K., Harris C.C., Koboldt D.C., Larson D. (2011). Recurrent mutations in the U2AF1 splicing factor in myelodysplastic syndromes. Nat. Genet..

[27] Papaemmanuil E., Cazzola M., Boultwood J., Malcovati L., Vyas P., Bowen D., Pellagatti A., Wainscoat J., Hellstrom-Lindberg E., Passerini C.G. (2011). SomaticSF3B1Mutation in Myelodysplasia with Ring Sideroblasts. N. Engl. J. Med..

[28] Yoshida K., Sanada M., Shiraishi Y., Nowak D., Nagata Y., Yamamoto R., Sato Y., Sato-Otsubo A., Kon A., Nagasaki M. (2011). Frequent pathway mutations of splicing machinery in myelodysplasia. Nature.

[29] Gozani O., Feld R., Reed R. (1996). Evidence that sequence-independent binding of highly conserved U2 snRNP proteins upstream of the branch site is required for assembly of spliceosomal complex A. Genes Dev..

[30] Haferlach T., Nagata Y., Grossmann V., Okuno Y., Bacher U., Nagae G., Schnittger S., Sanada M., Kon A., Alpermann T. (2014). Landscape of genetic lesions in 944 patients with myelodysplastic syndromes. Leukemia.

[31] Malcovati L., Papaemmanuil E., Bowen D.T., Boultwood J., Della Porta M.G., Pascutto C., Travaglino E., Groves M.J., Godfrey A.L., Ambaglio I. (2011). Clinical significance of SF3B1 mutations in myelodysplastic syndromes and myelodysplastic/myeloproliferative neoplasms. Blood.

[32] Papaemmanuil E., Gerstung M., Malcovati L., Tauro S., Gundem G., Van Loo P., Yoon C.J., Ellis P., Wedge D., Pellagatti A. (2013). Clinical and biological implications of driver mutations in myelodysplastic syndromes. Blood.

[33] Mortera-Blanco T., Dimitriou M., Woll P.S., Karimi M., Elvarsdottir E., Conte S., Tobiasson M., Jansson M., Douagi I., Moarii M. (2017). SF3B1-initiating mutations in MDS-RSs target lymphomyeloid hematopoietic stem cells. Blood.

[34] Malcovati L., Stevenson K., Papaemmanuil E., Neuberg D., Bejar R., Boultwood J., Bowen D.T., Campbell P.J., Ebert B.L., Fenaux P. (2020). SF3B1-mutant MDS as a distinct disease subtype: A proposal from the International Working Group for the Prognosis of MDS. Blood.

[35] Hahn C., Scott H.S. (2011). Spliceosome mutations in hematopoietic malignancies. Nat. Genet..

[36] Quesada V., Conde L., Villamor N., Ordóñez G.R., Jares P., Bassaganyas L., Ramsay A.J., Beà S., Pinyol M., Martínez-Trillos A. (2011). Exome sequencing identifies recurrent mutations of the splicing factor SF3B1 gene in chronic lymphocytic leukemia. Nat. Genet..

[37] Darman R.B., Seiler M., Agrawal A.A., Lim K.H., Peng S., Aird D., Bailey S.L., Bhavsar E.B., Chan B., Colla S. (2015). Cancer-Associated SF3B1 Hotspot Mutations Induce Cryptic 3′ Splice Site Selection through Use of a Different Branch Point. Cell Rep..

[38] DeBoever C., Ghia E.M., Shepard P.J., Rassenti L., Barrett C.L., Jepsen K., Jamieson C.H.M., Carson D., Kipps T.J., Frazer K.A. (2015). Transcriptome Sequencing Reveals Potential Mechanism of Cryptic 3′ Splice Site Selection in SF3B1-mutated Cancers. PLoS Comput. Biol..

[39] Dolatshad H., Pellagatti A., Fernandez-Mercado M., Yip B.H., Malcovati L., Attwood M., Przychodzen B., Sahgal N., Kanapin A., Lockstone H.E. (2015). Disruption of SF3B1 results in deregulated expression and splicing of key genes and pathways in myelodysplastic syndrome hematopoietic stem and progenitor cells. Leukemia.

[40] Dolatshad H., Pellagatti A., Liberante F., Llorian M., Repapi E., Steeples V., Roy S., Scifo L., Armstrong R., Shaw J. (2016). Cryptic splicing events in the iron transporter ABCB7 and other key target genes in SF3B1-mutant myelodysplastic syndromes. Leukemia.

[41] Kesarwani A.K., Ramirez O., Gupta A.K., Yang X., Murthy T., Minella A., Pillai M.M. (2017). Cancer-associated SF3B1 mutants recognize otherwise inaccessible cryptic 3′ splice sites within RNA secondary structures. Oncogene.

[42] Brogna S., Wen J. (2009). Nonsense-mediated mRNA decay (NMD) mechanisms. Nat. Struct. Mol. Biol..

[43] Kurosaki T., Popp M.W., Maquat L.E. (2019). Quality and quantity control of gene expression by nonsense-mediated mRNA decay. Nat. Rev. Mol. Cell Biol..

[44] Pellagatti A., Armstrong R.N., Steeples V., Sharma E., Repapi E., Singh S., Sanchi A., Radujkovic A., Horn P., Dolatshad H. (2018). Impact of spliceosome mutations on RNA splicing in myelodysplasia: Dysregulated genes/pathways and clinical associations. Blood.

[45] Shiozawa Y., Malcovati L., Galli’ A., Sato-Otsubo A., Kataoka K., Sato Y., Watatani Y., Suzuki H., Yoshizato T., Yoshida K. (2018). Aberrant splicing and defective mRNA production induced by somatic spliceosome mutations in myelodysplasia. Nat. Commun..

[46] Makishima H., Visconte V., Sakaguchi H., Jankowska A.M., Abu Kar S., Jerez A., Przychodzen B., Bupathi M., Guinta K., Afable M.G. (2012). Mutations in the spliceosome machinery, a novel and ubiquitous pathway in leukemogenesis. Blood.

[47] Obeng E.A., Chappell R.J., Seiler M., Chen M.C., Campagna D.R., Schmidt P.J., Schneider R.K., Lord A.M., Wang L., Gambe R.G. (2016). Physiologic Expression of Sf3b1(K700E) Causes Impaired Erythropoiesis, Aberrant Splicing, and Sensitivity to Therapeutic Spliceosome Modulation. Cancer Cell.

[48] Visconte V., Lindsley C., Berlyne D. (2015). Aplastic Anemia & MDS International Foundation (AA&MDSIF): Bone Marrow Failure Disease Scientific Symposium 2014. Leuk. Res..

[49] Visconte V., Rogers H.J., Singh J., Barnard J., Bupathi M., Traina F., McMahon J., Makishima H., Szpurka H., Jankowska A. (2012). SF3B1 haploinsufficiency leads to formation of ring sideroblasts in myelodysplastic syndromes. Blood.

[50] Mupo A., Seiler M., Sathiaseelan V., Pance A., Yang Y., Agrawal A.A., Iorio F., Bautista R., Pacharne S., Tzelepis K. (2017). Hemopoietic-specific Sf3b1-K700E knock-in mice display the splicing defect seen in human MDS but develop anemia without ring sideroblasts. Leukemia.

[51] Kaida D., Motoyoshi H., Tashiro E., Nojima T., Hagiwara M., Ishigami K., Watanabe H., Kitahara T., Yoshida T., Nakajima H. (2007). Spliceostatin A targets SF3b and inhibits both splicing and nuclear retention of pre-mRNA. Nat. Chem. Biol..

[52] Corrionero A., Miñana B., Valcárcel J. (2011). Reduced fidelity of branch point recognition and alternative splicing induced by the anti-tumor drug spliceostatin A. Genes Dev..

[53] Kotake Y., Sagane K., Owa T., Mimori-Kiyosue Y., Shimizu H., Uesugi M., Ishihama Y., Iwata M., Mizui Y. (2007). Splicing factor SF3b as a target of the antitumor natural product pladienolide. Nat. Chem. Biol..

[54] Fu X.-D., Maniatis T. (1990). Factor required for mammalian spliceosome assembly is localized to discrete regions in the nucleus. Nature.

[55] Hou H.-A., Tsai C.-H., Lin C.-C., Chou W.-C., Kuo Y.-Y., Liu C.-Y., Tseng M.-H., Peng Y.-L., Liu M.-C., Liu C.-W. (2018). Incorporation of mutations in five genes in the revised International Prognostic Scoring System can improve risk stratification in the patients with myelodysplastic syndrome. Blood Cancer J..

[56] Chen M., Manley J.L. (2009). Mechanisms of alternative splicing regulation: Insights from molecular and genomics approaches. Nat. Rev. Mol. Cell Biol..

[57] Kim E., Ilagan J.O., Liang Y., Daubner G.M., Lee S., Ramakrishnan A., Li Y., Chung Y.R., Micol J.-B., Murphy M.E. (2015). SRSF2 Mutations Contribute to Myelodysplasia by Mutant-Specific Effects on Exon Recognition. Cancer Cell.

[58] Komeno Y., Huang Y.J., Qiu J., Lin L., Xu Y., Zhou Y., Chen L., Monterroza D.D., Li H., DeKelver R.C. (2015). SRSF2 Is Essential for Hematopoiesis, and Its Myelodysplastic Syndrome-Related Mutations Dysregulate Alternative Pre-mRNA Splicing. Mol. Cell. Biol..

[59] Zhang J., Lieu Y.K., Ali A., Penson A., Reggio K.S., Rabadan R., Raza A., Mukherjee S., Manley J. (2015). Disease-associated mutation in SRSF2 misregulates splicing by altering RNA-binding affinities. Proc. Natl. Acad. Sci. USA.

[60] Masaki S., Ikeda S., Hata A., Shiozawa Y., Kon A., Ogawa S., Suzuki K., Hakuno F., Takahashi S.-I., Kataoka N. (2019). Myelodysplastic Syndrome-Associated SRSF2 Mutations Cause Splicing Changes by Altering Binding Motif Sequences. Front. Genet..

[61] Ernst T., Chase A.J., Score J., Hidalgo-Curtis C.E., Bryant C., Jones A.V., Waghorn K., Zoi K., Ross F.M., Reiter A. (2010). Inactivating mutations of the histone methyltransferase gene EZH2 in myeloid disorders. Nat. Genet..

[62] Kon A., Yamazaki S., Nannya Y., Kataoka K., Ota Y., Nakagawa M.M., Yoshida K., Shiozawa Y., Morita M., Yoshizato T. (2018). Physiological Srsf2 P95H expression causes impaired hematopoietic stem cell functions and aberrant RNA splicing in mice. Blood.

[63] Shirahata-Adachi M., Iriyama C., Tomita A., Suzuki Y., Shimada K., Kiyoi H. (2017). Altered EZH2 splicing and expression is associated with impaired histone H3 lysine 27 tri-Methylation in myelodysplastic syndrome. Leuk. Res..

[64] Yoshimi A., Lin K.-T., Wiseman D., Rahman M.A., Pastore A., Wang B., Lee S., Micol J.-B., Zhang X.J., De Botton S. (2019). Coordinated alterations in RNA splicing and epigenetic regulation drive leukaemogenesis. Nature.

[65] Chen L., Chen J.-Y., Huang Y.-J., Gu Y., Qiu J., Qian H., Shao C., Zhang X., Hu J., Li H. (2018). The Augmented R-Loop Is a Unifying Mechanism for Myelodysplastic Syndromes Induced by High-Risk Splicing Factor Mutations. Mol. Cell.

[66] Nguyen H.D., Leong W.Y., Li W., Reddy P.N., Sullivan J.D., Walter M.J., Zou L., Graubert T.A. (2018). Spliceosome Mutations Induce R Loop-Associated Sensitivity to ATR Inhibition in Myelodysplastic Syndromes. Cancer Res..

[67] Ruskin B., Zamore P.D., Green M.R. (1988). A factor, U2AF, is required for U2 snRNP binding and splicing complex assembly. Cell.

[68] Zamore P., Patton J.G., Green M.R. (1992). Cloning and domain structure of the mammalian splicing factor U2AF. Nature.

[69] Thol F., Kade S., Schlarmann C., Löffeld P., Morgan M., Krauter J., Wlodarski M., Kölking B., Wichmann M., Görlich K. (2012). Frequency and prognostic impact of mutations in SRSF2, U2AF1, and ZRSR2 in patients with myelodysplastic syndromes. Blood.

[70] Hou H.-A., Liu C.-Y., Kuo Y.-Y., Chou W.-C., Tsai C., Lin C.-C., Lin L.-I., Tseng M.-H., Chiang Y.-C., Liu M.-C. (2016). Splicing factor mutations predict poor prognosis in patients with de novo acute myeloid leukemia. Oncotarget.

[71] Przychodzen B., Jerez A., Guinta K., Sekeres M.A., Padgett R., Maciejewski J.P., Makishima H. (2013). Patterns of missplicing due to somatic U2AF1 mutations in myeloid neoplasms. Blood.

[72] Brooks A.N., Choi P., De Waal L., Sharifnia T., Imielinski M., Saksena G., Pedamallu C.S., Sivachenko A., Rosenberg M., Chmielecki J. (2014). A Pan-Cancer Analysis of Transcriptome Changes Associated with Somatic Mutations in U2AF1 Reveals Commonly Altered Splicing Events. PLoS ONE.

[73] Ilagan J.O., Ramakrishnan A., Hayes B.J., Murphy M.E., Zebari A.S., Bradley P., Bradley R. (2015). U2AF1mutations alter splice site recognition in hematological malignancies. Genome Res..

[74] Okeyo-Owuor T., White B.S., Chatrikhi R., Mohan D.R., Kim S., Griffith M., Ding L., Ketkar-Kulkarni S., Hundal J., Laird K.M. (2015). U2AF1 mutations alter sequence specificity of pre-mRNA binding and splicing. Leukemia.

[75] Shao C., Yang B., Wu T., Huang J., Tang P., Zhou Y., Zhou J., Qiu J., Jiang L., Li H. (2014). Mechanisms for U2AF to define 3′ splice sites and regulate alternative splicing in the human genome. Nat. Struct. Mol. Biol..

[76] Shirai C.L., Ley J.N., White B.S., Kim S., Tibbitts J., Shao J., Ndonwi M., Wadugu B., Duncavage E.J., Okeyo-Owuor T. (2015). Mutant U2AF1 Expression Alters Hematopoiesis and Pre-mRNA Splicing In Vivo. Cancer Cell.

[77] Yip B.H., Steeples V., Repapi E., Armstrong R.N., Llorian M., Roy S., Shaw J., Dolatshad H., Taylor S., Verma A. (2017). The U2AF1S34F mutation induces lineage-specific splicing alterations in myelodysplastic syndromes. J. Clin. Investig..

[78] Smith M.A., Choudhary G.S., Pellagatti A., Choi K., Bolanos L., Bhagat T.D., Gordon-Mitchell S., Von Ahrens D., Pradhan K., Steeples V. (2019). U2AF1 mutations induce oncogenic IRAK4 isoforms and activate innate immune pathways in myeloid malignancies. Nat. Cell Biol..

[79] Yoshida H., Park S.-Y., Sakashita G., Nariai Y., Kuwasako K., Muto Y., Urano T., Obayashi E. (2020). Elucidation of the aberrant 3′ splice site selection by cancer-associated mutations on the U2AF1. Nat. Commun..

[80] Warnasooriya C., Feeney C.F., Laird K.M., Ermolenko D.N., Kielkopf C.L. (2020). A splice site-sensing conformational switch in U2AF2 is modulated by U2AF1 and its recurrent myelodysplasia-associated mutation. Nucleic Acids Res..

[81] Shen H., Zheng X., Luecke S., Green M.R. (2010). The U2AF35-related protein Urp contacts the 3′ splice site to promote U12-type intron splicing and the second step of U2-type intron splicing. Genes Dev..

[82] Madan V., Kanojia D., Li J., Okamoto R., Sato-Otsubo A., Kohlmann A., Sanada M., Grossmann V., Sundaresan J., Shiraishi Y. (2015). Aberrant splicing of U12-type introns is the hallmark of ZRSR2 mutant myelodysplastic syndrome. Nat. Commun..

[83] Inoue D., Polaski J.T., Taylor J., Castel P., Chen S., Kobayashi S., Hogg S.J., Hayashi Y., Pineda J.M.B., El Marabti E. (2021). Minor intron retention drives clonal hematopoietic disorders and diverse cancer predisposition. Nat. Genet..

[84] MacRae A., Mayerle M., Hrabeta-Robinson E., Chalkley R., Guthrie C., Burlingame A.L., Jurica M.S. (2018). Prp8 positioning of U5 snRNA is linked to 5′ splice site recognition. RNA.

[85] Kurtovic-Kozaric A., Przychodzen B., Singh J.A., Konarska M.M., Clemente M.J., Otrock Z.K., Nakashima M., Hsi E.D., Yoshida K., Shiraishi Y. (2015). PRPF8 defects cause missplicing in myeloid malignancies. Leukemia.

[86] Fortes P., Cortes D.B., Fornerod M., Rigaut G., Raymond W., Séraphin B., Mattaj I.W. (1999). Luc7p, a novel yeast U1 snRNP protein with a role in 5′ splice site recognition. Genes Dev..

[87] Maciejewski J.P., Padgett R.A., Brown A.L., Müller-Tidow C. (2017). DDX41-related myeloid neoplasia. Semin. Hematol..

[88] Ogawa S. (2014). Splicing factor mutations in AML. Blood.

[89] Zhu L., Zhang Y., Zhang W., Yang S., Chen J.-Q., Tian D. (2009). Patterns of exon-intron architecture variation of genes in eukaryotic genomes. BMC Genom..

[90] Izaurralde E., Lewis J., McGuigan C., Jankowska M., Darzynkiewicz E., Mattaj I.W. (1994). A nuclear cap binding protein complex involved in pre-mRNA splicing. Cell.

[91] Kataoka N., Ohno M., Moda I., Shimura Y. (1995). Identification of the factors that interact with NCBP, an 80 kDa nuclear cap binding protein. Nucleic Acids Res..

[92] Ohno M., Kataoka N., Shimura Y. (1990). A nuclear cap binding protein from HeLa cells. Nucleic Acids Res..

[93] Niwa M., Rose S.D., Berget S.M. (1990). In vitro polyadenylation is stimulated by the presence of an upstream intron. Genes Dev..

